# Dengue virus downregulates TNFR1- and TLR3-stimulated NF-κB activation by targeting RIPK1

**DOI:** 10.3389/fcimb.2022.926036

**Published:** 2022-10-14

**Authors:** Darshika J. Udawatte, Diane M. Lang, Jeffrey R. Currier, Carey L. Medin, Alan L. Rothman

**Affiliations:** ^1^ Institute for Immunology and Informatics, Department of Cell and Molecular Biology, University of Rhode Island, Providence, RI, United States; ^2^ Viral Diseases Branch, Walter Reed Army Institute of Research, Silver Spring, MD, United States

**Keywords:** dengue virus, NS3, ripk1, NF-κB, necroptosis

## Abstract

Dengue virus (DENV) infection is the most prevalent arthropod-borne virus disease and is endemic in more than 100 countries. Several DENV proteins have been shown to target crucial human host proteins to evade innate immune responses and establish a productive infection. Here we report that the DENV NS3 protein targets RIPK1 (Receptor Interacting Protein Kinase I), a central mediator of inflammation and cell death, and decreases intracellular RIPK1 levels during DENV infection. The interaction of NS3 with RIPK1 results in the inhibition of NF-κB activation in response to TNFR or TLR3 stimulation. Also, we observed that the effects of NS3 on RIPK1 were independent of NS3 protease activity. Our data demonstrate a novel mechanism by which DENV suppresses normal cellular functions to evade host innate immune responses

## Introduction

Dengue is the most prevalent arthropod-borne viral disease, with an estimated 400 million infections occurring annually (Bhatt et al., 2013). Infected individuals may develop severe symptoms leading to dengue hemorrhagic fever (DHF), dengue shock syndrome (DSS), and even death. The causative agent of dengue, dengue virus (DENV), is known to infect dendritic cells, macrophages, monocytes, B cells, T cells, hepatocytes, epithelial and endothelial cells (King et al., 1999; Martina et al., 2009; Silveira et al., 2018). Dengue disease is thought to result both from direct viral effects as well as host immune responses to infection (Mathew and Rothman, 2008; Martina et al., 2009). Effective antiviral drugs and vaccines are not available to treat dengue, which emphasizes the importance of better understanding DENV-host interactions (Low et al., 2017). DENV refers to a group of four closely related positive-sense, single-stranded RNA viruses or serotypes (DENV 1, 2, 3, 4), belonging to the *Flaviviridae* family. Upon entry into the host cell, the viral genome is translated into a single polypeptide, which is cleaved into its individual structural (capsid, PrM, and envelope) and non-structural proteins (NS1, NS2A, NS2B, NS3, NS4A, NS4B, NS5) by viral and host proteases (Clum et al., 1997). The DENV protease is a complex of NS2B and NS3 proteins. NS2B is a small hydrophobic protein that acts as a cofactor for NS3 proteolytic activity (Falgout et al., 1991). The N-terminal 180 acids of NS3 contain a serine protease domain (Wengler et al., 1991), while the remainder of NS3 (amino acids 180 to 618) contains an RNA helicase domain and a NTPase motif (Xu et al., 2005; Luo et al., 2008). In addition to their roles in viral RNA replication and packaging, several DENV proteins are known to interact with numerous host proteins and interfere with host antiviral immune responses. For example, the DENV protease NS2B3 interacts with and cleaves STING and downregulates RLR signaling (Aguirre et al., 2012). The DENV protease also cleaves the mitochondrial proteins MFN1 and MFN2 and thereby impairs mitochondrial dynamics as well as efficient RLR signaling (Yu et al., 2015).

RIPK1 has emerged as an important regulator that controls multiple cellular pathways involved in inflammation and cell death (Ofengeim and Yuan, 2013). RIPK1 contributes to innate immune responses to bacterial and viral infections by inducing cell death (apoptosis or necroptosis) or by inducing inflammatory signaling (Upton and Kaiser, 2017). Both kinase and scaffolding functions of RIPK1 are important for signaling. Activation of death receptors such as TNFR1 leads to the formation of a multi-protein complex including RIPK1, complex I, which facilitates the activation of NF-κB and MAPK pathways that promote cell survival by inducing anti-apoptotic gene expression and induce proinflammatory cytokine gene expression (Hsu et al., 1996). The scaffolding function of RIPK1 is critical for NF-κB activation. RIPK1 is also involved in mediating TRIF-dependent NF-κB activation in response to TLR3 and TLR4 stimulation (Meylan et al., 2004; Cusson-Hermance et al., 2005). RIPK1 can drive extrinsic apoptosis by activating caspase-8 *via* the formation of a distinct multi-protein complex, complex IIa (Micheau and Tschopp, 2003), and can drive necroptosis through formation of a complex with RIPK3 (complex IIb) leading to phosphorylation of the pseudokinase mixed lineage kinase domain-like (MLKL) protein (Cho et al., 2009; He et al., 2009). RIPK1 also has been shown to regulate IRF3 activation downstream of RIG-I/MDA-5 in response to RNA viruses (Rajput et al., 2011). This central role of RIPK1 makes it an ideal target for inhibition by viruses, as has been shown for HIV-1 (Wagner et al., 2015), human Rhinovirus 3C (Croft et al., 2018), and Epstein-Barr virus (Liu et al., 2018). However, no association between RIPK1 and DENV infection has been reported to date.

In the present study, we observed that RIPK1 protein levels decreased over time during DENV infection. Plasmid expression of DENV NS3, which lacks protease activity, was sufficient to decrease intracellular RIPK1 levels. Using co-immunoprecipitation and Western blot analysis, we observed that DENV NS3 physically interacted with RIPK1. DENV infection and expression of NS3 inhibited the TNFα- and poly (I:C)-stimulated activation of NF-κB. Furthermore, MLKL phosphorylation in response to TNFα was suppressed by DENV infection. We hypothesize that inhibition of RIPK1-mediated regulation of cell death may represent an additional mechanism to promote DENV replication.

## Materials and methods

### Cell culture and virus infection

Huh7, HEK 293T cells stably expressing TLR3 (generous gifts from Dr. Kate Fitzgerald), HEK 293T (Dharmacon, Inc.), and Vero (obtained from American Type Culture Collection (ATCC)) cells were maintained in Dulbecco’s modified minimal essential medium and HepG2 (ATCC) cells were maintained in Eagle’s Minimum Essential Medium, both supplemented with 10% heat-inactivated fetal bovine serum (Sigma-Aldrich), 1% penicillin-streptomycin (Sigma-Aldrich), 1% non-essential amino acids (Lonza) and 1% L-Glutamine solution (Sigma-Aldrich). U937 cells stably expressing DC-SIGN (a generous gift from Dr. Anuja Mathew) were maintained in Roswell Park Memorial Institute (RPMI) medium supplemented with 10% heat-inactivated fetal bovine serum (Sigma-Aldrich), 1% penicillin-streptomycin (Sigma-Aldrich), 1% non-essential amino acids (Lonza), and 1% L-Glutamine solution (Sigma-Aldrich). All cells were incubated in a humidified chamber at 37°C and 5% CO2. DENV2 strains DENV2 16681 and NGC were originally obtained from ATCC or the Walter Reed Army Institute of Research and were passaged in C6/36 cells (ATCC). Virus titers were determined by immunostained plaque assay on Vero cells (Liu et al., 2012; Medin et al., 2015).

### Generation of MoDCs

Peripheral blood mononuclear cells (PBMCs) were isolated from buffy coat of healthy donors obtained from the Oklahoma Blood Institute (Oklahoma City, OK) by density gradient centrifugation on Ficoll-Paque Premium (GE Healthcare). Monocytes were isolated from PBMCs by positive selection using CD14+ microbeads (Miltenyi Biotec). Monocytes were cultured in RPMI 1640 medium supplemented with 10% heat-inactivated FBS (Sigma-Aldrich), 1% penicillin- streptomycin (Sigma-Aldrich), 50 ng/mL human interleukin-4 (IL-4) and 160 ng/mL human granulocyte-macrophage colony-stimulating factor (GM-CSF) for 5 days to generate monocyte-derived dendritic cells (MoDCs). Fresh medium with cytokines was added at day 3, and cells were infected with DENV2 at day 5.

### Antibodies and reagents

The following primary antibodies were used for co-immunoprecipitation and Western blot experiments: anti-Flag-HRP (sc-166355, Santa Cruz Biotechnology) anti-Flag (F1804, Sigma-Aldrich), mouse anti-RIPK3 (sc-374639, Santa Cruz Biotechnology), anti-β-actin-HRP (ab20272, Abcam), anti-V5 tag (ab27671, Abcam), anti-V5-HRP (R961-25, Invitrogen), anti-GAPDH-HRP (MA515738) anti-DENV NS3 (GTX124252, GeneTex), anti-DENV NS4B (GTX103349, GeneTex), anti-DENV NS1 (GTX103346, GeneTex), anti-DENV NS2B (GTX124246, GeneTex) anti-RIPK1 (D94C12, Cell Signaling Technology), anti-LC3B (2775S, Cell Signaling Technology), anti-p-MLKL (91689S, Cell Signaling Technology). Secondary antibodies used were goat anti-rabbit-HRP (32260, Thermo Scientific) and anti-mouse-HRP (sc516102, Santa Cruz Biotechnology). The following reagents were used for inhibitor treatment experiments: NH_4_Cl (A9434, Sigma-Aldrich), Chloroquine (C6628, Sigma-Aldrich), MG132 (NC9038428, Fisher Scientific), z-VAD-FMK (SC-3067, Santa Cruz Biotechnology) and DMSO (BP231-100, Fisher Scientific).

### Western blot analysis

Cells were lysed in RIPA buffer freshly supplemented with protease and phosphatase inhibitor cocktail (Sigma-Aldrich). Lysates were incubated on ice for 30 min with vortexing every 5 min and centrifuged at 10,000 rpm for 5 min at 4°C. Supernatants were used as total cell lysates. Protein concentrations were determined using Pierce BCA Protein Assay kit (Thermo Fisher Scientific) following manufacturer’s instructions and measured using an Envision plate reader (PerkinElmer). BOLT LDS Sample Buffer and Reducing Agent (Thermo Fisher Scientific) were added to cell lysates at a final concentration of 1x and samples were denatured at 70°C for 10 min. Proteins were separated on 4–12% or 8% BOLT Bis-Tris Plus gel (Thermo Fisher Scientific) and transferred onto nitrocellulose membranes using the Trans-Blot^®^ TurboTM RTA Mini Nitrocellulose Transfer kit (Bio-Rad). Membranes were blocked in 5% milk in phosphate buffered saline with 0.1% Tween 20 for 1 h at room temperature (rt) followed by incubation with the appropriate primary antibody overnight at 4°C. Membranes were washed and incubated with the appropriate secondary antibody diluted in blocking buffer for 1 hour at rt. For β-actin and GAPDH analysis, membranes were stained for 1 hour with corresponding antibodies. Detection of blots was done using Amersham ECL Select Western Blotting Detection Reagent (GE Healthcare Life Sciences) following the manufacturer’s guidelines and images were captured with ChemiDocTM XRS+ System (Bio-Rad). Detection and quantification of band intensities were performed using Image Lab 5.1 (Bio-Rad).

### Co-immunoprecipitation

Cells were lysed with RIPA buffer containing a cocktail of protease and phosphatase inhibitors. Protein G dynabeads (Thermo Fisher Scientific) were incubated with either mouse anti-Flag (Sigma-Aldrich), mouse anti-V5 (abcam) or mouse anti-NS3 (Genetex) for 1 hr at rt. Antibody-bead complexes were washed with antibody binding and washing buffer (Thermo Fisher Scientific). Cell lysates (100μg) were incubated with antibody-coated beads for 30 min at RT with rotation. Bead-antibody-antigen complexes were washed 4 times with antibody binding and washing buffer followed by the washing buffer provided (Thermo Fisher Scientific). The immunocomplex was then eluted under denaturing conditions using elution buffer (Thermo Fisher Scientific), dithiothreitol (Thermo Scientific) and Bolt sample buffer (Life Technologies) according to the manufacturer’s recommendations, and then examined by Western blot analysis using the indicated antibodies.

### Plasmids and transfections

Plasmids pNS4B5-eGFP and pNS2B3-V5 have previously been described (Medin et al., 2015). pNS2B3-S135A was created by site-directed mutagenesis (Quik Change II site-directed mutagenesis kit, Agilent Technologies) of the pNS2B3-V5 plasmid. Plasmid pNS3-V5 was generated by PCR amplification of the NS3 coding sequence from cDNA of DENV2 strain New Guinea C obtained from BEI Resources, which was cloned into the Gateway pcDNA-DEST40 vector (ThermoFisher Scientific) following the manufacturer’s protocol. Primer sequences were: forward primer NS2B3-S135A-V5: 5’-GCT GTA TCT CTG GAC TTT TCT CCT GGA ACG GCC GGC TCT CCA ATT ATC GAC-3’; reverse primer NS2B3-S135A: 5’-GTC GAT AAT TGG AGA GCC GGC CGT TCC AGG AGA AAA GTC CAG AGA TAC AGC-3’; forward primer NS3-V5; 5’-GGG GAC AAG TTT GTA CAA AAA AGC AGG CTG CCA CCA TGG CTG GAG TAT TGT GGG ATG TC-3’; reverse primer NS3-V5; GGG GAC CAC TTT GTA CAA GAA AGC TGG GTT CTT TCT TCC AGC TGC AAA CTC-3’. The plasmid sequences were verified by DNA sequencing. Plasmid pNS2B3-pro contains a codon-optimized sequence encoding the full length of NS2B and the protease domain (residues 1-185) of NS3 derived from the S16681 isolate of DENV2 (Genbank: NC001474), which was synthesized by GenScript and cloned into in the pcDNA3.1/Zeo vector. Plasmids expressing Flag-RIPK1 and RIPK3-GFP were obtained from Addgene. The luciferase reporter constructs containing a tandem repeat of the consensus binding site for the transcription factor NF-κB, and a *Renilla reniformis* luciferase were obtained from Strategene. Transfection of cells with plasmids was performed using GeneJuice^®^ Transfection Reagent (EMD Millipore), according to the manufacturer’s instructions.

### RT-qPCR

Total RNA was extracted from cell pellets using the RNeasy Mini Kit (Qiagen). Quantitative RT- PCR was performed using iTaq Universal probes 1 step kit (Biorad) or iTaq Universal SYBR Green 1 step kit (Biorad) on a Bio-Rad CFX machine, and gene expression was normalized to β-actin expression level. Taqman gene expression assays (RIPK1) were purchased from Thermofisher Scientific. DENV primers were purchased from Integrated DNA Technology (IDT) and RT-qPCR was performed to detect relative DENV RNA in cell lysates (Sadon et al., 2008).

### Luciferase reporter assays

HEK 293T cells were plated in 96 well plates and were co-transfected with a NF-κB luciferase reporter, a Renilla luciferase reporter, and a plasmid encoding Flag-tagged RIPK1 using GeneJuice^®^ Transfection Reagent (EMD Millipore), according to the manufacturer’s protocol. Co-transfected cells were infected with DENV2 (NGC, MOI=3) and were cultured for 72hrs or transfected with plasmids expressing DENV NS3, NS2B3, NS2B3-S135A, or NS1 and cultured for 48hrs. Cells were then stimulated with hTNFα (50ng/ml, Peprotech) for the final 6hrs. Luciferase activity was quantified using the dual Glo luciferase assay system (Promega). HEK 293T cells stably expressing TLR3 were co-transfected and infected with DENV2 or overexpressed DENV NS3, NS2B3, NS2B3-S135A, or NS1 as described above and were stimulated with poly I:C (20µg/ml) for the final 24hrs. At the end of the treatment, luciferase activity was measured using the dual Glo luciferase assay system (Promega). The luciferase signal for each well was normalized by the Renilla luciferase signal.

### Necroptosis assay

U937-DC-SIGN cells (6 x 10^5^) were plated in a 6 well plate and infected with DENV2 (MOI=6) for 48hrs. Cells were treated with 50µM Nec-1 (Sigma Aldrich), 20µM zVAD-FMK (Santa Cruz Biotechnology), 50ng/ml TNFα (Peprotech), and 10µM Smac mimetic (Fisher Scientific) for 6 hours. Cells were harvested by centrifugation at 1600rpm for 5 minutes and lysed with RIPA buffer supplemented with protease inhibitors. Cell lysates were analyzed by Western blot.

### Statistical analysis

The statistical analyses were performed using one-way analysis of variance (ANOVA) or the unpaired, two-tailed, Student’s t-test, with Shapiro-Wilk and Brown-Forsythe tests for normality and equal variance. P values were calculated using Graphpad Prism 8 program. Differences were considered statistically significant at P values less than 0.05.

## Results

### RIPK1 protein levels decrease with DENV infection

**Figure 1:**
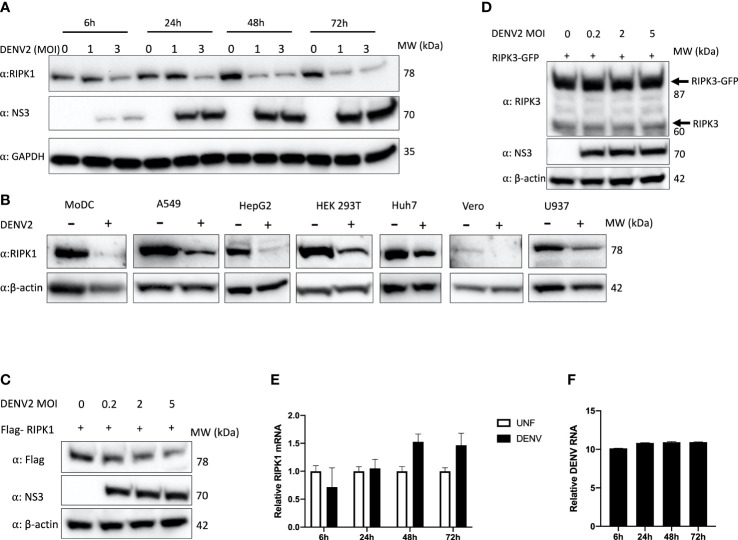
Endogenous and overexpressed RIPK1 decreases during DENV infection. **(A)** Time course study of DENV2 infection in human Huh7 cells with MOI of 1 and 3 for the indicated hours. Cell lysates were harvested for western blot analysis using the indicated antibodies. **(B)** Western blot analysis of RIPK1 in indicated cells infected with DENV2 (MOI=2) and cultured for 48hrs. β-actin was used as a loading control. **(C)** Western blot analysis of overexpressed RIPK1 and **(D)** RIPK3 followed by DENV2 infection in HEK 293T cells at 48h post-infection. **(E)** RT-qPCR analysis of total RNA isolated from Huh7 cells infected with DENV2 (MOI=2) for **(E)** RIPK1 or **(F)** intracellular DENV RNA for the indicated time points. UNF=Uninfected. Data are mean ± SD (n=3 experiments). Western blots are representative data from a minimum of 3 independent experiments (panels **A–D**).

DENV is known to infect hepatocytes and the liver is one of the major organs affected during DHF (Samanta, 2015). We tested the effect of DENV infection on the human hepatoma cell line, Huh7. A time-course analysis of DENV2 infection revealed a reduction in intracellular RIPK1 protein concomitant with the expression of DENV proteins, beginning as early as 6h post-infection (**Figure 1A**). In order to see if this was a general effect, we infected cell lines originated from different human tissues with DENV2 as well as monocyte-derived dendritic cells (MoDC), which is a relevant primary cell system (Ho et al., 2001). We observed a significant decrease in endogenous RIPK1 protein levels with DENV infection in all the cells tested (**Figure 1B**). Overexpressed Flag-tagged RIPK1 was also decreased in a dose-response manner in DENV-infected HEK 293T cells (**Figure 1C**). This effect was specific for RIPK1 since endogenous or overexpressed RIPK3 levels were not affected by DENV infection (**Figure 1D**).

Next, we investigated if DENV inhibited RIPK1 at a transcriptional level. A time-course study was done with DENV2 infection in Huh7 cells. We did not see a decrease in RIPK1 transcript levels, indicating that the downregulation of RIPK1 occurred at the post-transcriptional level (**Figures 1E, F**).

### RIPK1 is reduced by DENV NS3 protein

**Figure 2:**
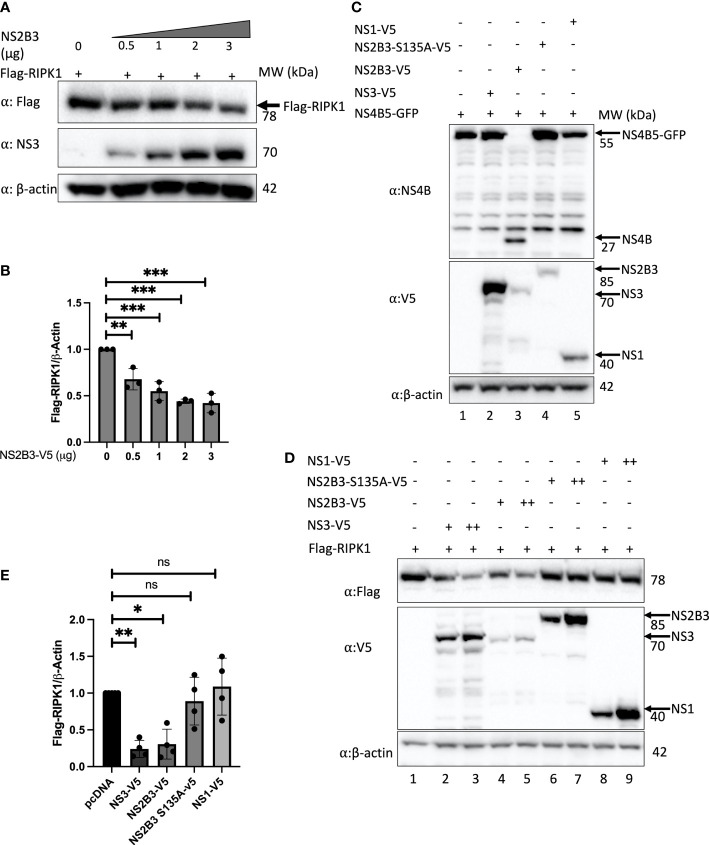
DENV NS3 decreases RIPK1 protein levels. **(A)** Western blot analysis of HEK 293T cells co-transfected with Flag-RIPK1 and increasing concentrations of a plasmid expressing the DENV NS2B3. **(B)** Densitometric analysis of Flag-RIPK1 band intensities normalized to β-actin levels. **(C)** Western blot analysis of HEK293T cells co-transfected with NS4B5-GFP and indicated plasmids at 48hrs post-transfection. **(D)**Western blot analysis of HEK 293T cells co-transfected with Flag-RIPK1 and 1μg (+) or 2.5μg (++) of indicated plasmids. **(E)** Densitometric analysis of Flag-RIPK1 band intensities normalized to β-actin levels. Results are representative of at least three independent experiments and are expressed as mean ± SD from three biological replicates. (One-way ANOVA, *P < 0.05, **P < 0.01, ***P < 0.001). ns, not significant.

RIPK1 is known to be targeted by several viral proteases to counteract its functions and enhance viral replication (Wagner et al., 2015; Croft et al., 2018). Therefore, to evaluate the mechanism of RIPK1 reduction during DENV infection, we tested whether expression of the DENV NS2B3 protease complex was sufficient to reduce RIPK1 levels. We co-transfected a Flag-tagged RIPK1 and a plasmid encoding DENV NS2B3. Previous studies with the NS2B3 plasmid shows that it efficiently self-cleaves the NS2B3-V5 product (85kDa) into NS3-V5 (70kDa) and NS2B (15kDa) (Medin et al., 2015). We observed a dose response in RIPK1 reduction with the expression of DENV NS2B3 (**Figures 2A, B**). However, we did not detect the accumulation of a cleaved product of RIPK1 using antibodies targeting either the C-terminal or N-terminal segment of RIPK1 (data not shown).

Next, to determine if the protease activity of NS2B3 was important for this effect, we adopted two strategies. First, we created a catalytically inactive protease mutant, NS2B3-S135A (Yu et al., 2006). Since it lacks protease activity it is unable to self-cleave and is detected as an 85kDa fusion protein on Western blot (**Figure 2C**). Second, we cloned the full-length NS3 coding sequence without NS2B. The NS3 plasmid yielded a stronger band with anti-V5 staining at the predicted size (70kDa) than the NS2B3 plasmid (**Figure 2C**). Additional fainter bands both larger and smaller than the expected size were also visible; these may reflect post-translational processing but were not studied further. Co-transfection of these constructs with a plasmid expressing a 55kDa fusion protein, NS4B5-GFP, which is a target for the DENV protease (Medin et al., 2015) confirmed the lack of protease activity for both NS2B3-S135A (**Figure 2C** lane 4) and NS3 alone (**Figure 2C** lane 2), whereas the active DENV NS2B3 plasmid cleaved the NS4B5-GFP fusion protein to yield NS4B, detected by the appearance of a new band at the expected size of 27kDa. (Other bands in **Figure 2C** were also seen in untransfected cell lysates stained with this rabbit polyclonal antibody.) A significant decrease in Flag-RIPK1 was detected with overexpression of NS2B3 and NS3 alone (**Figure 2D** lanes 2-5 & **Figure 2E**), whereas Flag-RIPK1 levels were not affected by the expression of DENV NS2B3-S135A or NS1 (**Figure 2D** lanes 6-9 & **Figure 2E**). Since the results with expression of NS3 alone confirm that protease activity is not required for the decrease in RIPK1, the lack of a similar effect with expression of NS2B3-S135A suggests that cleavage between NS2B and NS3 may be required for NS3 to exert this effect.

### RIPK1 physically interacts with DENV NS3

**Figure 3:**
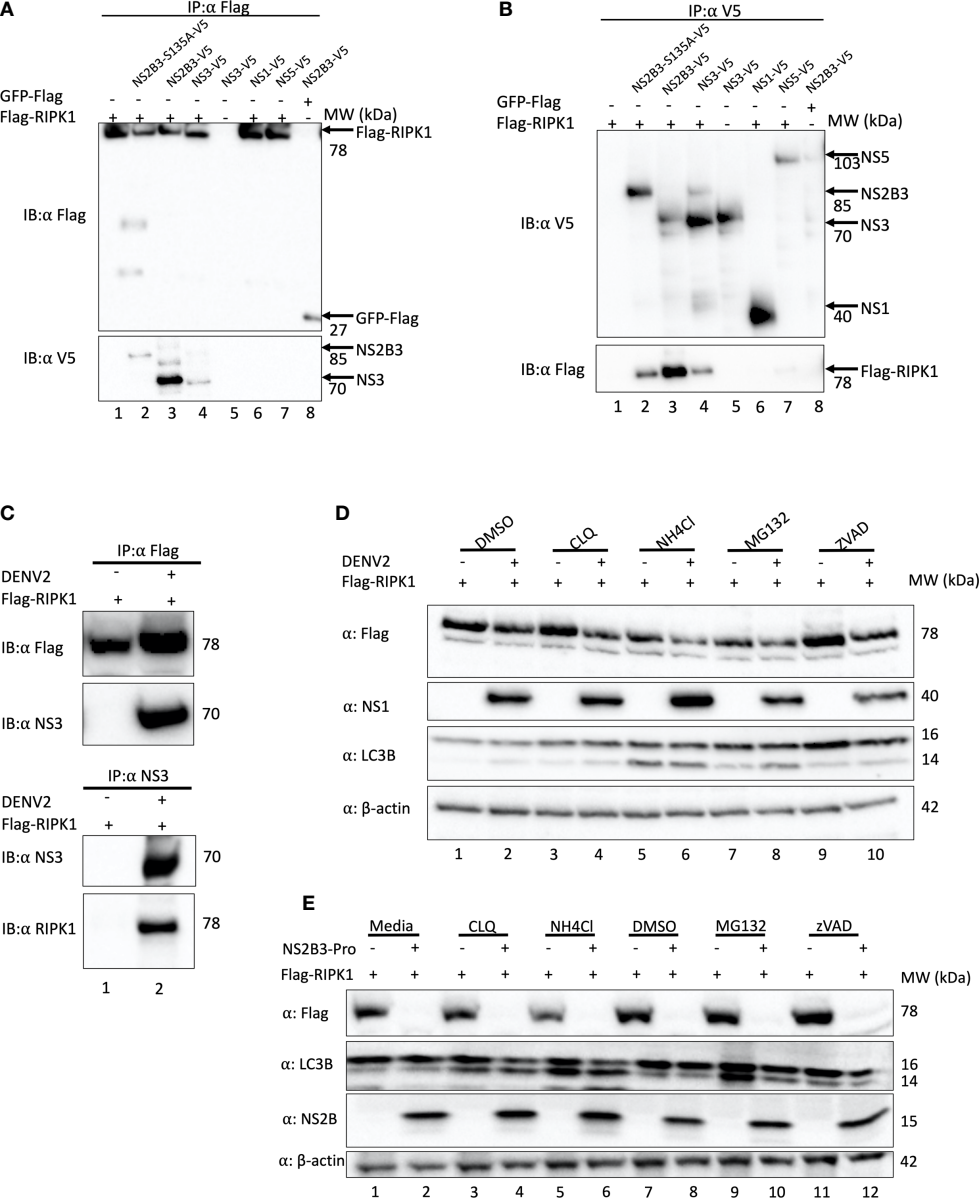
DENV NS3 binds to RIPK1 but does not mediate its degradation *via* proteasomal or lysosomal pathways. **(A, B)** Co-immunoprecipitation analyses of HEK 293T cells transiently overexpressing Flag-RIPK1 and indicated plasmids using α-Flag **(A)** or α-V5 **(B)** for pull down. **(C)** Co-immunoprecipitation analyses of HEK 293T cells transfected with Flag-RIPK1 and mock (media) or DENV2 (MOI=1) infected and cultured for 48hrs. **(D)** Western blot analysis of HEK293T cells infected with DENV2 for 48hrs and treated with Chloroquine (CLQ, 10μM), NH_4_Cl (15mM), MG132 (2.5μM), ZVAD (10μM) or DMSO for 6hrs and analyzed by indicated antibodies. β-actin is used as a loading control. **(E)** Co-expression of Flag-RIPK1 and DENV NS2B3-pro for 40hrs followed by treatment with indicated inhibitors for 6hrs and analyzed by western blot using indicated antibodies. Results are representative of at least three independent experiments. IP, immunoprecipitation; IB, immunoblot.

To evaluate if the reduction in RIPK1 is due to a direct interaction of NS3 with RIPK1, we next performed co-immunoprecipitation experiments. HEK 293T cells were co-transfected with a plasmid expressing Flag-tagged RIPK1 and plasmids expressing either NS3 lacking the NS2B domain, wild type (WT) DENV NS2B3 or NS2B3-S135A. Plasmids expressing Flag tagged GFP or DENV NS1 and NS5 proteins served as controls. DENV NS3, WT NS2B3, NS2B3-S135A, but not NS1 or NS5, co-immunoprecipitated with RIPK1 when pulled down with anti-Flag (**Figure 3A**, lanes 2, 3, 4). The reciprocal immunoprecipitation using anti-V5 antibodies confirmed this interaction (**Figure 3B**, lanes 2, 3, 4). NS3 protease activity was not required for the interaction with RIPK1, because neither lack of the NS2B domain nor the S135A mutation prevented NS3 interacting with RIPK1. We then validated this interaction of RIPK1 and NS3 in the context of DENV infection. HEK 293T cells transiently expressing Flag-RIPK1 were infected with DENV2 for 48hrs, then RIPK1 was immunoprecipitated using an anti-Flag antibody. We detected DENV NS3 bound to RIPK1 by Western blot analysis (**Figure 3C** lane 2). We performed the reciprocal immunoprecipitation using an anti-DENV NS3 antibody and detected RIPK1 bound to DENV-NS3 by Western blot analysis, verifying the interaction (**Figure 3C** lane 2).

DENV has been shown to increase protein degradation *via* the autophagy-lysosome pathway. DENV protease co-factor NS2B interacted with and promoted autophagosome-mediated degradation of cGAS, which is a key component in the DNA sensing pathway (Aguirre et al., 2017). DENV NS2B3 interacted with and promoted lysosome mediated degradation of Nfr2, a component in the antioxidant response (Ferrari et al., 2020). Furthermore, negative regulation of RIPK1 by caspase 8 is well known. During activation of death receptors, active caspase 8 cleaves RIPK1 in the intermediate domain at Asp 324 (Lin et al., 1999).

To test if DENV NS3 also promotes RIPK1 for lysosomal or proteasomal degradation, HEK293T cells were treated with the lysosome inhibitors NH_4_Cl and chloroquine (CLQ), or the proteasome inhibitor MG132 following DENV2 infection and transfection of the Flag-RIPK. ZVAD-FMK, a pan caspase inhibitor, was used to test the role of caspases in the decrease in RIPK1 caused by DENV NS3. After 6 hours of inhibitor treatments, the cell lysates were analyzed by Western blotting. As previously observed, RIPK1 levels decreased with DENV2 infection (**Figure 3D**). However, the RIPK1 levels did not recover with the inhibition of either the proteasome or the lysosome pathways during DENV infection (**Figure 3D** lanes 4,6,8). Chloroquine and NH_4_Cl treatment clearly increased the LC3-II levels indicating efficient inhibition of lysosomal function (**Figure 3D** lanes 4,5,6). Caspase inhibition by ZVAD increased Flag-RIPK1 levels in uninfected cells yet the RIPK1 levels were still suppressed by DENV2 (**Figure 3D** lanes 9,10). Taken together these data suggest that RIPK1 is not subjected to these cellular degradation mechanisms by DENV NS3.

To confirm this result, we obtained a plasmid containing a codon-optimized sequence of the DENV2 NS2B and NS3 protease domain (see Methods). This plasmid caused a profound decrease in RIPK1 levels (**Figure 3E**). However, we still observed no significant lessening of this effect by treatment with proteasomal, lysosomal or caspase inhibitors (**Figure 3E**).

### DENV impairs TNFR- and TLR3-induced NF-κB activation

**Figure 4:**
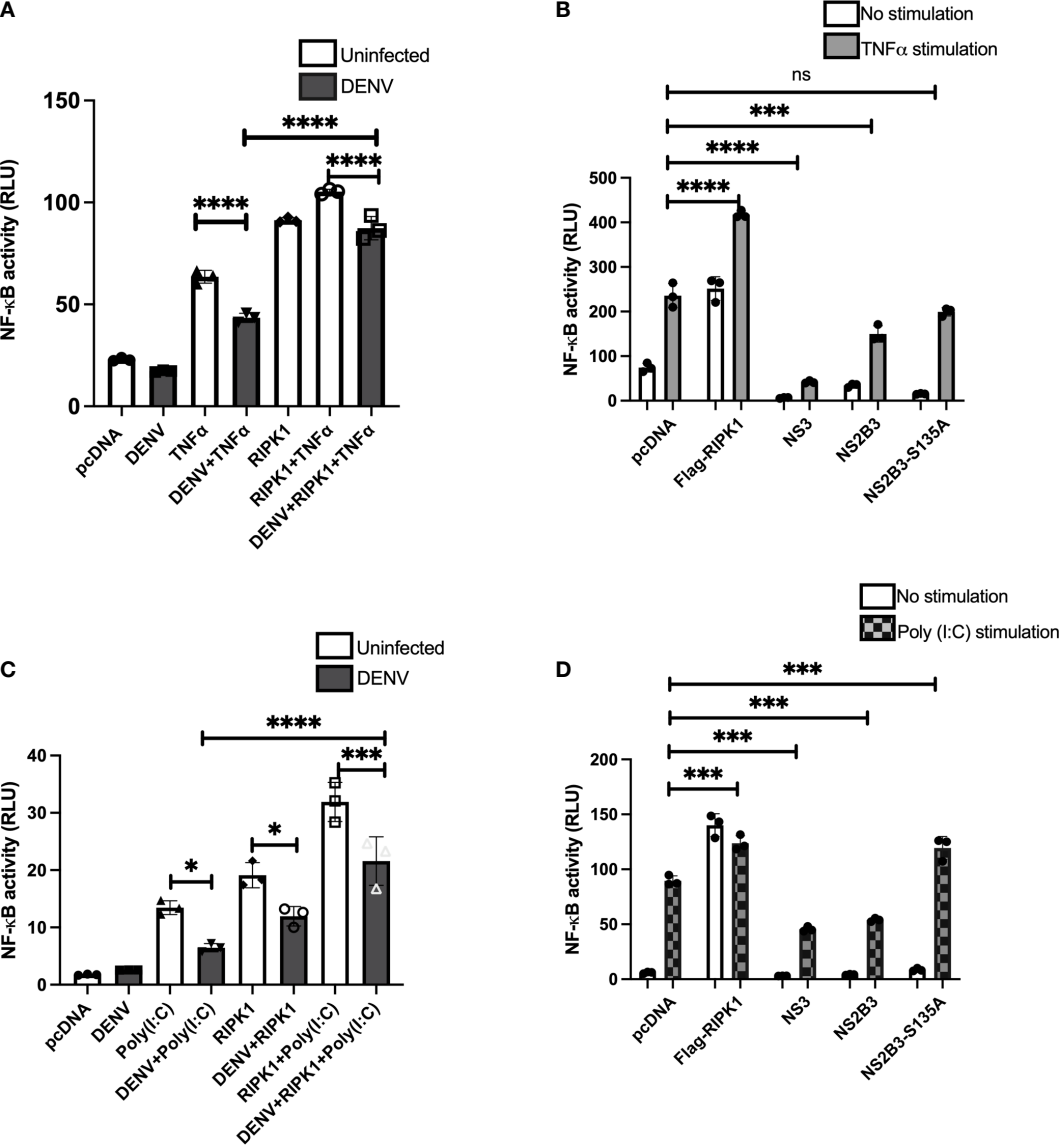
TNFα- and poly(I:C)-driven NF-κB activation is impaired during DENV infection and expression of DENV NS3. **(A)** HEK 293T cells were co-transfected with NF-κB luciferase reporter, Renilla luciferase reporter and Flag-RIPK1 followed by DENV2 infection (MOI=3). Following TNFα (50ng/ml) treatment for 6hrs, cells were lysed, and luciferase activity was measured at 72hrs post-infection. Open bars=uninfected. Closed bars=DENV infected. **(B)** HEK 293T cells were co-transfected with NF-κB luciferase reporter, Renilla luciferase reporter, and Flag-RIPK1 along with NS3, WT NS2B3 or NS2B3-S135A. Luciferase activity was detected at 48hrs post-transfection following 6hrs of TNFα (50ng/ml) treatment. **(C)** NF-κB luciferase activity was assessed in HEK 293T-TLR3 cells as in **(A)** and cells were stimulated with poly (I:C) 20µg/ml for 24hrs before cell lysis. Open bars=uninfected. Closed bars=DENV infected. **(D)** HEK 293T-TLR3 cells were co-transfected with NF-κB luciferase reporter, Renilla luciferase reporter, and Flag-RIPK1 along with NS3, WT NS2B3 or NS2B3-S135A. Luciferase activity was detected at 48hrs post-transfection following 24hrs of poly (I:C) 20µg/ml treatment. Results are representative of at least three independent experiments and are expressed as mean ± SD from three replicate wells. (One-way ANOVA, *P < 0.05, ***P < 0.001, ****P < 0.0001). ns, not significant.

RIPK1 is an adapter molecule downstream of TNFR1 and TLRs and plays an important role in downstream signaling leading to NF-κB activation (Micheau and Tschopp, 2003; Cusson-Hermance et al., 2005). Considering the observation of NS3-RIPK1 interaction and subsequent decrease in RIPK1 levels, we wanted to learn the effect of DENV in these signaling pathways. We assessed NF-κB luciferase activity in response to TNFα stimulation with DENV infection in HEK 293T cells. NF-κB luciferase activity was significantly reduced with DENV infection, and this was restored by overexpression of RIPK1 (**Figure 4A**). A similar pattern of inhibition of NF-κB luciferase activity in response to TNFα stimulation was detected by constructs expressing DENV NS3 or NS2B3 (**Figure 4B**). Expression of the protease-negative NS2B3-S135A mutant did not have a significant effect.

We also examined the effect of DENV infection on NF-κB activation in response to TLR3 stimulation. HEK 293T cells stably expressing TLR3 were transfected with the luciferase reporter plasmids with or without the Flag-RIPK1 plasmid and then infected with DENV (MOI=3). They were further stimulated with a synthetic viral RNA mimic, poly I:C, for 24hrs and luciferase activity was measured. As with TNFR1 signaling, DENV infection impaired poly I:C-induced NF-κB activation relative to uninfected cells (**Figure 4C**). Also similar to the results with TNFR1 stimulation, overexpression of RIPK1 partially overcame the inhibition of poly I:C-induced NF-κB activation by DENV. NF-κB activity following stimulation with poly I:C was also inhibited with overexpression of DENV NS3 or NS2B3 (**Figure 4D**). Overexpression of the NS2B3-S135A mutant did not inhibit TLR3 signaling, in fact, there was a very small but reproducible increase in poly I:C induced NF-κB activation (**Figure 4D**). These results demonstrate that DENV infection and expression of NS3 protein impairs NF-κB activation downstream of both TNFR1 and TLR3 by decreasing RIPK1 protein levels.

### DENV suppresses TSZ-induced MLKL phosphorylation

**Figure 5:**
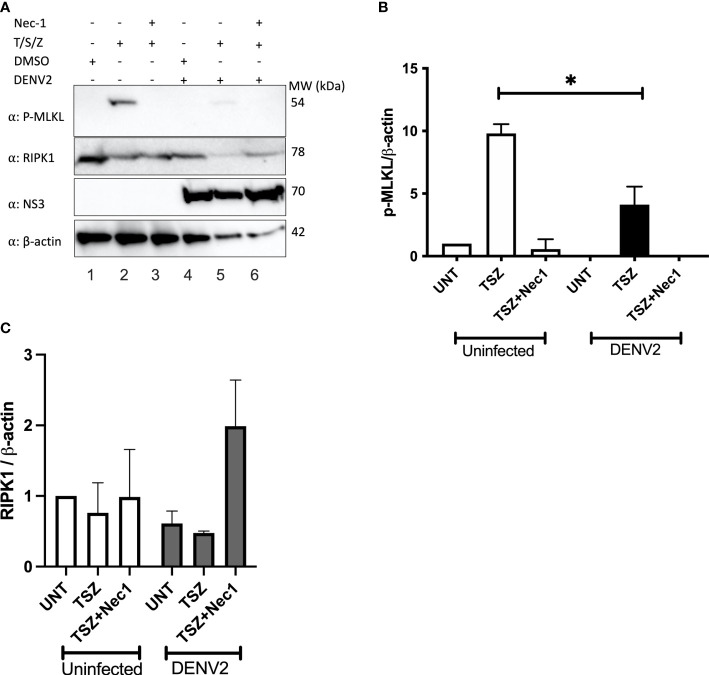
T/S/Z-induced MLKL phosphorylation is suppressed by DENV. **(A)** Western blot analysis to detect phosphorylated MLKL (p-MLKL), RIPK1, DENV NS3 and β-actin in cell lysates from DENV infected or uninfected U937-DC-SIGN cells. Cells were treated with T/S/Z, T/S/Z +Nec-1 or the vehicle control, DMSO. T-TNFα (50ng/ml), S-Smac mimetic (10μM), Z-ZVAD-FMK (20μM), Necrostatin-1 (Nec-1/50μM). **(B)** Densitometric analysis of phosphorylated MLKL and **(C)** endogenous RIPK1 band intensities normalized to β-actin levels. Results are representative of at least two independent experiments and are expressed as mean ± SD from two biological replicates. (t test, *P < 0.05).

Programmed necrosis or necroptosis has been recognized as an antiviral cell death mechanism (Vandenabeele et al., 2010). Since RIPK1 is a critical component in necroptosis induction downstream of TNFR1, TLR3, TLR4 and ZBP1, viruses have evolved proteins to target and decrease RIPK1 levels or block RIPK1 signaling (Mack et al., 2008; Ofengeim and Yuan, 2013; Humphries et al., 2015; Wagner et al., 2015). Numerous cell lines have been shown to undergo necroptosis in response to TNFα, Smac mimetic and ZVAD (T/S/Z) stimulation (He et al., 2009; Cai et al., 2014), and we used U937 cells stably expressing DC-SIGN for our assays. Necroptosis can be pharmacologically inhibited by the RIPK1 inhibitor necrostatin-1 (Degterev et al., 2008). To investigate the effect of DENV on host cell necroptosis we treated DENV-infected and uninfected cells with necroptosis inducers T/S/Z and/or necrostatin-1. Since MLKL phosphorylation at T357/358 is critical for the induction of necroptosis we then performed immunoblot analysis of p-MLKL. T/S/Z treatment induced increased levels of p-MLKL in uninfected cells which was significantly suppressed by treatment with necrostatin-1 (**Figures 5A** lane 2, **5B**). In contrast, p-MLKL was only faintly detected in DENV-infected cells following T/S/Z treatment (**Figures 5A** lane 5, **5B**). These data indicate that DENV inhibits MLKL phosphorylation upon T/S/Z stimulation, a marker of necroptosis, potentially due to the decrease in RIPK1 levels (**Figure 5C**).

## Discussion

RIPK1 is a critical regulator in innate immune responses, hence it has emerged as an attractive target for inhibition by numerous virus families (Udawatte and Rothman, 2021). RIPK1 plays a protective role against flavivirus infections. Mice expressing kinase dead RIPK1 (RIPK1^KD/KD^) were more susceptible to West Nile virus infections than WT mice (Daniels et al., 2017). Furthermore, RIPK1^KD/KD^ mice had higher viral titers than controls upon infection with Zika virus (Daniels et al., 2019). To date, a role for RIPK1 during DENV infection has not been established. In this study we show that intracellular RIPK1 levels are reduced by DENV infection *in vitro*, and that this reduction in intracellular RIPK1 levels is associated with inhibition of RIPK1 signaling downstream of TNFR1 (NF-κB activation and necroptosis induction) and TLR3 (NF-κB activation). We further show that these effects are caused by expression of the DENV NS3 protein. The effect was not directly dependent on NS3 protease activity or on the presence of NS2B, however, cleavage of NS2B from NS3 was necessary for the decrease in RIPK1 and for inhibition of RIPK1 signaling. These findings add RIPK1 to the list of host proteins targeted by the DENV NS3 (Yu et al., 2015; Stabell et al., 2018).

RIPK1 signaling is known to be inhibited by numerous viral proteins. HIV-1 protease (PR) has been shown to cleave RIPK1. Thus, HIV-1 PR suppress RIPK1-mediated NF-κB activation and necroptosis (Wagner et al., 2015). MCMV protein M45 is known to block RIPK1 signaling by interacting with RIPK1 and blocking RIPK1 interacting with other signaling proteins (Mack et al., 2008; Upton et al., 2008). DENV NS3 protein expression was sufficient to decrease RIPK1 levels. The protease activity did not play a role in decreasing RIPK1 levels, as shown by the significant decrease in RIPK1 with overexpression of a NS3 construct lacking the cofactor NS2B. However, a protease inactive mutant with NS2B intact interacted with RIPK1 but did not affect RIPK1 levels. Therefore, the presence of NS2B may be preventing the RIPK1 decrease by an unknown mechanism. NS2B3-S135A was also shown to interact with STING even though it did not affect STING levels (Aguirre et al., 2012). We were not able to clearly define a mechanism by which DENV NS3 promotes the decrease in RIPK1 levels. It has been reported that DENV NS3 blocks RIG-I and 14-3-3ε signaling in a proteolysis-independent manner (Chan and Gack, 2016). DENV NS2B3 was shown to promote Nrf2 for lysosomal degradation by a protease-independent mechanism (Ferrari et al., 2020). DENV NS2B was also shown to promote cGAS for lysosomal degradation. Our data suggest that neither proteasomal nor lysosomal degradation was responsible for the decrease in RIPK1 levels by DENV infection or NS3 expression (**Figures 3D, E**).

Increased serum TNFα levels have been reported in patients with dengue (Yadav et al., 1991). The interaction of TNFα and TNFR1 results in the formation of complex I involving RIPK1 leading to NF-κB activation (Aggarwal, 2003). RIPK1 also signals for NF-κB activation downstream of TLR3 (Meylan et al., 2004). NF-κB transcription factors regulate many genes involved in immune responses. Therefore, signaling leading to NF-κB activation serves as an anti-viral immune mechanism. Viruses inhibit NF-κB pathway activation to evade these host immune and inflammatory responses (Zhao et al., 2015). DENV infection was shown to block NF-κB activation downstream of TLR signaling resulting in downregulated cytokine production (Chang et al., 2012). Similar studies in Huh7 and monocyte-derived macrophages showed that DENV infection altered cellular responses to TNFα stimulation (Wati et al., 2011). However, the mechanism by which DENV caused these altered responses had not been defined. We demonstrated that DENV infection diminished NF-κB activation in response to TNFα or poly I:C stimulation and that this effect could be partially overcome by overexpression of RIPK1. We also observed that expression of DENV NS3 caused a similar inhibition of NF-κB activation in response to TNFα or polyI:C. These results indicate that the levels of RIPK1 are important for regulating upstream signals leading to NF-κB activation. A detailed understanding of the mechanism by which the RIPK1-NS3 interaction affects the formation of signaling complexes downstream of TNFR1 and TLR3 will require further investigation.

Liver injury is a common finding in DENV infection and can lead to organ failure. Apoptosis of hepatocytes following DENV infection is believed to contribute to this phenomenon (Samanta, 2015). Recent findings show a protective role of RIPK1 in TNFα-mediated apoptosis in hepatocytes (Filliol et al., 2016; Farooq et al., 2019). RIPK1 knockout mice showed enhanced sensitivity to TNFα-mediated apoptosis and inflammation. Induction of NF-κB target gene transcripts was also suppressed in these mice (Filliol et al., 2016). In light of these reports, we speculate that loss of RIPK1 during DENV infection may sensitize hepatocytes to cell death and inflammation, which could contribute to liver injury in DHF.

RIPK1-mediated necroptosis has been described as a general strategy to inhibit viral replication (Huang et al., 2015; Shubina et al., 2020; Udawatte and Rothman, 2021). Several viruses are known to inhibit necroptosis by targeting RIPK1, and different mechanisms have been described (Udawatte and Rothman, 2021). To our knowledge ours is the first report that DENV infection can inhibit T/S/Z-induced phosphorylation of MLKL, an indicator of necroptosis. Modulating cell death pathways would be advantageous for non-cytolytic viruses, as inhibiting or delaying cell death could increase the number of progeny viruses, and this effect might contribute to disease pathogenesis.

This is the first description of DENV targeting RIPK1, and the first study to link inhibition of NF-κB signaling by DENV to targeting of RIPK1 by the DENV NS3 protein. Furthermore, this is the first evidence of suppression of necroptosis by DENV. These findings open new research directions on the interplay of DENV with various emerging functions of RIPK1. Further studies are required to shed light on cell- and context-dependent effects of RIPK1 reduction *in vivo* during DENV infection. Finally, we believe that these data expand knowledge of strategies used by DENV to evade innate immune responses.

## Data availability statement

The original contributions presented in the study are included in the article/supplementary material, further inquiries can be directed to the corresponding author/s.

## Author contributions

DU, CM, and AR contributed to the conception and design of the experiments. JC contributed critical reagents. DU and DL performed the experiments and collected the data. DU analyzed the data, prepared the final figures, and wrote the first draft of the manuscript. CM and AR obtained funding for the experiments. All authors contributed to the article and approved the submitted version.

## Funding

Research reported in this publication was supported by the National Institute of Allergy and Infectious Diseases of the (NIH) under award number P01AI034533. The funder played no role in the study. The content is solely the responsibility of the authors and does not necessarily represent the official views of the (NIH).

## Acknowledgments

We would like to thank Dr. Katherine Fitzgerald for generously providing HEK 293T cells stably expressing TLR3, and Dr. Drishya Diwaker for technical assistance.

## Conflict of interest

The authors declare that the research was conducted in the absence of any commercial or financial relationships that could be construed as a potential conflict of interest.

## Publisher’s note

All claims expressed in this article are solely those of the authors and do not necessarily represent those of their affiliated organizations, or those of the publisher, the editors and the reviewers. Any product that may be evaluated in this article, or claim that may be made by its manufacturer, is not guaranteed or endorsed by the publisher.
